# Spätzle Homolog-Mediated Toll-Like Pathway Regulates Innate Immune Responses to Maintain the Homeostasis of Gut Microbiota in the Red Palm Weevil, *Rhynchophorus ferrugineus* Olivier (Coleoptera: Dryophthoridae)

**DOI:** 10.3389/fmicb.2020.00846

**Published:** 2020-05-25

**Authors:** Abrar Muhammad, Prosper Habineza, Xinghong Wang, Rong Xiao, Tianliang Ji, Youming Hou, Zhanghong Shi

**Affiliations:** ^1^State Key Laboratory of Ecological Pest Control for Fujian and Taiwan Crops, Fujian Agriculture and Forestry University, Fuzhou, China; ^2^Fujian Provincial Key Laboratory of Insect Ecology, College of Plant Protection, Fujian Agriculture and Forestry University, Fuzhou, China; ^3^Guizhou Institute of Biology, Guizhou Academy of Sciences, Guiyang, China

**Keywords:** *Rhynchophorus ferrugineus*, Spätzle, toll pathway, antimicrobial peptides, gut microbiota

## Abstract

Spätzle (Spz) is a dimeric ligand that responds to the Gram-positive bacterial or fungal infection by binding Toll receptors to induce the secretion of antimicrobial peptides. However, whether the Toll-like signaling pathway mediates the innate immunity of *Rhynchophorus ferrugineus* to modulate the homeostasis of gut microbiota has not been determined. In this study, we found that a Spz homolog, RfSpätzle, is a secretory protein comprising a signal peptide and a conservative Spz domain. RT-qPCR analysis revealed that *RfSpätzle* was significantly induced to be expressed in the fat body and gut by the systemic and oral infection with pathogenic microbes. The expression levels of two antimicrobial peptide genes, *RfColeoptericin* and *RfCecropin*, were downregulated significantly by *RfSpätzle* knockdown, indicating that their secretion is under the regulation of the *RfSpätzle*-mediated signaling pathway. After being challenged by pathogenic microbes, the cumulative mortality rate of *RfSpätzle*-silenced individuals was drastically increased as compared to that of the controls. Further analysis indicated that these larvae possessed the diminished antibacterial activity. Moreover, *RfSpätzle* knockdown altered the relative abundance of gut bacteria at the phylum and family levels. Taken together, these findings suggest that *RfSpätzle* is involved in RPW immunity to confer protection and maintain the homeostasis of gut microbiota by mediating the production of antimicrobial peptides.

## Introduction

Spätzle (Spz), a dimeric ligand of the Toll receptor, binds the Toll receptor to initiate the secretion of antimicrobial peptides via activating the immune signaling pathway (Lemaitre and Hoffmann, 2007). In invertebrates, genomic studies have revealed that different invertebrate species have various numbers of Spz gene copies. For example, *Drosophila melanogaster* has six *Spz* isoforms (*Dm*Spz1–6) (Parker et al., 2001), while *Aedes aegypti* (Shin et al., 2006), black tiger shrimp *Peneaus monodon* (Boonrawd et al., 2017), and white shrimp *Litopenaeus vannamei* (Wang et al., 2012) have three Spz proteins. However, only one *Spz* gene, *MrSpz*, was identified from the freshwater prawn *Macrobrachium rosenbergii* (Vaniksampanna et al., 2019). Furthermore, previous evidence indicated that *Bm*Spz1 of *Bombyx mori* (Wang et al., 2007), *Ms*Spz1 in *Manduca sexta* (An et al., 2010), and *Ap*Spz in oak silkworm *Antheraea pernyi* (Sun et al., 2016) were markedly induced by microbial infection to activate their immune responses. In *D*. *melanogaster*, the Spz-mediated Toll pathway has been well determined to mediate the synthesis of antimicrobial peptides, such as *drosomycin* (Weber et al., 2003; Valanne et al., 2011; Stokes et al., 2015), and activate the cellular immunity (Hultmark, 2003).

Insects harbor diverse species of symbiotic microorganisms in their guts that strongly affect host physiological traits, including nutrition metabolism (Wong et al., 2014; Janssen and Kersten, 2015; Muhammad et al., 2017; Hebineza et al., 2019), detoxification (Engel and Moran, 2013), protection from natural enemies (Kim et al., 2015; Park et al., 2018), growth and development (Blatch et al., 2010; Wong et al., 2014; Hebineza et al., 2019), mating and foraging behavior (Ami et al., 2010), and gut homeostasis (Engel and Moran, 2013; Douglas, 2015). However, the exact mechanism by which these insect hosts modulate the intensity of gut immunity to maintain the homeostasis of gut microbiota is still poorly understood outside *D. melanogaster*. In *D. melanogaster*, it is well-known that gut epithelial cells can secrete antimicrobial peptides and reactive oxygen species (ROS), which provide colonization resistance to non-commensal bacteria (Ha et al., 2005; Ryu et al., 2008; Lee et al., 2017). The production of these two immune effectors is under the control of the IMD (immune deficiency) pathway and dual oxidase (DUOX) signaling system, respectively (Lemaitre and Hoffmann, 2007; Ryu et al., 2008; Lee et al., 2017; Lin et al., 2018). Moreover, the IMD pathway has been shown to control the excessive proliferation of residential gut microbiota, as the IMD mutant exhibited an increased gut bacterial load (Guo et al., 2014). Intestinal immunity is regulated to maintain the homeostasis of the gut microbiota. For instance, a number of recognition proteins, including *PGRP-LB*, *PGRP-SC1a*, *PGRP-SC1b*, and *PGRP-SC2*, negatively regulate the IMD signaling pathway due to their amidase activity, degrading gut bacteria-derived peptidoglycan to avoid the excessive production of immune effectors in gut epithelial cells (Bischoff et al., 2006; Zaidman-Rémy et al., 2006; Paredes et al., 2011; Guo et al., 2014; Dawadi et al., 2018; Maire et al., 2018, 2019). Additionally, negative regulation can also be achieved by the regulator PIRK, interacting with *PGRP-LC*, *PGRP-LE*, and *Imd* to prevent excessive activation of the IMD signaling pathway (Aggarwal and Silverman, 2008).

To the best of our knowledge, the interactions between the gut microbiota and host immune system in many insects are far from well understood. Red palm weevil (RPW), *Rhynchophorus ferrugineus* (Olivier) (Coleoptera: Dryophthoridae), is an immensely destructive pest for palm plants in China and other tropical countries (Ju et al., 2011; Shi et al., 2014; Al-Dosary et al., 2016). The RPW gut is colonized by a complex gut bacterial community that is involved in the degradation of plant polysaccharides to impact host nutrition metabolism (Jia et al., 2013; Tagliavia et al., 2014; Montagna et al., 2015; Muhammad et al., 2017; Hebineza et al., 2019). Interestingly, RPW also houses an intracellular symbiont, *Nardonella*, within a specialized organ, the bacteriome (Hosokawa et al., 2015). *Nardonella* provides its host with tyrosine, which is used for cuticle synthesis and hardening (Anbutsu et al., 2017). Recently, we found that RfPGRP-LB acts as a negative immunity regulator to inhibit the chronic activation of the immune response by degrading peptidoglycan (Dawadi et al., 2018). It has also been found that weevil *pgrp-lb* prevents endosymbiont-released immunological molecules from escaping the bacteriocytes to chronically activate host systemic immunity (Maire et al., 2019). Furthermore, an NF-κB-like transcription factor, RfRelish, has been demonstrated to mediate the intestinal immunity to modulate the homeostasis of the RPW gut microbiota (Xiao et al., 2019). However, the role of the Spz-mediated Toll-like pathway in RPW immunity has not been determined. In this study, a Spz homolog, *RfSpätzle*, was characterized and its role in modulating the composition of the RPW gut microbiota was determined. Our evidence indicated that the *RfSpätzle*-mediated Toll signaling pathway regulates the synthesis of antimicrobial peptides to confer the protection against microbial infection and modulates the proportion of RPW gut bacteria.

## Materials and Methods

### Insect Rearing

The RPW laboratory population was established and maintained by adults trapped in the Pingtan District (119°32′ E, 25°31′ N) of Fuzhou city, Fujian Province and Jinshan campus of Fujian Agriculture and Forestry University (119°30′ E, 26°08′ N). RPW individuals were fed with sugarcane stems in an incubator (Saifu ZRX-260, Ninbo Experimental Instrument Co. Ltd., China) at 27 ± 1°C, 75 ± 5% relative humidity, and a photoperiod of 24 h dark for larvae and 12 h light/12 h dark for adults (Muhammad et al., 2017).

### Full-Length cDNA Cloning and Sequence Analysis of *RfSpätzle*

The fourth instar RPW larvae were dissected in a sterilized dish plate containing 2 ml of phosphate buffered saline (PBS, NaCl 137 mM, KCl 2.7 mM, Na_2_HPO_4_ 10 mM, K_2_HPO_4_ 2 mM, pH 7.2). Three guts were pooled as replicates and homogenized with a tissue lyser (Ningbo Scientz BioTech. Company Limited, China). Total RNA was extracted with TRIzol Reagent (Invitrogen, Carlsbad, CA, United States) following the manufacturers’ instructions. RNA concentration and integrity were determined with NanoDrop2000 (Thermo Fisher Scientific Inc., Waltham, MA) and 1% agarose gel electrophoresis, respectively. The cDNA template was prepared with the TransScript^®^ All-in-One First Strand cDNA Synthesis Kit (Takara Bio Inc., Dalian, China). The core sequence of *RfSpätzle* was cloned with a pair of specific primers (Table 1). The 25-μl PCR mix consisted of 1 μl of cDNA, 12.5 μl of 2 × TaqPCR mix, 1 μl each of forward and reverse primer (10 μM) and 9.5 μl of RNase-free water. Thermal conditions were set as follows: the initial denaturation at 95°C for 2 min followed by 35 cycles at 95°C for 30 s, 60°C for 30 s, 72°C for 1 min, and a final extension at 68°C for 7 min. PCR products were confirmed by 1% agarose gel electrophoresis. The full cDNA length of *RfSpätzle* was obtained through rapid amplification of cDNA ends (RACE), which was completed by nested PCR with a SMARTer^®^ RACE kit (Takara Bio Inc., Dalian, China) according to manufacturer guidelines. PCR products were purified with an EasyPure^®^ Quick Gel Extraction Kit (TransGen Biotech, Beijing, China). Subsequently, the purified PCR products were ligated into the p*EASY*^®^ -T1 cloning vector (TransGen Biotech, Beijing, China). The full cDNA sequence of *RfSpätzle* was analyzed with ExPASY tool^1^ to determine its open reading frame (ORF). Conservative domains of RfSpätzle were predicted with running the SMART program^2^.

**TABLE 1:** Primer sequences used in this study.

Primer	Sequence (5′–3′)
**Confirmatory PCR**	
*RfSpätzle*-F	GAAGAGGACGAAGACGACAA
*RfSpätzle*-R	GAACGTGTCGCTGTAGAGGT
**For cDNA cloning using RACE analysis**	
3 R outer (*RfSpätzle*-F)	GCGAACATCCCGACCATTAT
5 R inner (*RfSpätzle*-R)	TCACCACGTACATCCAGTTTC
3 R inner (*RfSpätzle*-F)	CGTGGCATTGAACGGTTATTC
5 R outer (*RfSpätzle*-R)	GTATTTCGACGTGAGAGGATGT
**For dsRNA synthesis**	
dsRNA-SPZ-F	**TAATACGACTCACTATAGGG**CCGTGT
	ACATTCCAAAGCCT
dsRNA-SPZ-R	**TAATACGACTCACTATAGGG**GATTCG
	GGCATATTCACCAC
dsRNA-eGFP-F	**TAATACGACTCACTATAGGG**AGACAG TGCTTCAGCCGCTAC
dsRNA-eGFP-R	**TAATACGACTCACTATAGGG**AGAGTT CACCTTGATGCCGTTC
**For RT-qPCR**	
*RfSpätzle*-F	ACCCGTGTACATTCCAAAGC
*RfSpätzle* -R	TAGTTGGATTGCACGAGCTG
*RfAttacin-F*	TGGTTCTGGTGCCCAAGTGA
*RfAttacin-R*	GCCATAACGATTCTTGTTGGAGTA
*RfCecropin-F*	CAGAAGCTGGTTGGTTGAAGA
*RfCecropin-R*	GCAACACCGACATAACCCTGA
*RfDefensin-F*	TTCGCCAAACTTATCCTCGTG
*RfDefensin-R*	GGGTGCTTCGTTATCAACTTCC
*RfColeoptericin-F*	TCGTGGTTTCTACCATGTTCACT
*RfColeoptericin-R*	TCAGCTAAAACCTGATCTTGGA
*Rf*β*-actin-F*	CCAAGGGAGCCAAGCAATT
*Rf*β*-actin-R*	CGCTGATGCCCCTATGTATGT

*The bold letters represent the T7 promoter which is added to the specific primers.*

### Multiple Sequence Alignments and Phylogenetic Analysis of *RfSpätzle*

BLASTX^3^ was run to find the annotated Spz sequences from other insect species for the following multiple alignments. These Spz sequences were retrieved from the NCBI database. Multiple sequence alignments were performed with the Clustal Omega Program^4^. Phylogenetic analysis was completed with MEGA 5.05.

### Expression Profile of *RfSpätzle* Across Different Tissues and Its Transcriptional Response to Microbial Infection

The healthy fourth instar larvae were dissected for collecting various tissues, including head, epidermis, hemolymph, fat body and gut, to determine the expression profile of *RfSpätzle*. Three larvae were dissected as a replicate and each treatment comprised four replicates. Total RNA was extracted from the tissues with TRIzol Reagent (Invitrogen, Carlsbad, CA, United States). Total RNA (1 μg) was used to synthesize cDNA with a Thermo Fisher Scientific Verso cDNA Kit (Thermo Fisher Scientific, United States). To detect the transcript abundance of *RfSpätzle*, RT-qPCR was performed with FastStart Universal SYBR Green Master (Roche, United States) with a 20-μl reaction system, including 1 μl of cDNA and 125 nM specific primers. *Rf*β*-Actin* was employed as the internal reference gene. The qPCR reactions were completed with the following thermal cycling conditions: denaturation at 95°C for 10 min, 40 cycles of 95°C for 15 s, amplification at 60°C for 1 min, and with a dissociation step. The 2^–ΔΔ*Ct*^ method was used to calculate the relative expression level of our target genes.

To investigate the potential role of *RfSpätzle* in RPW immunity, its transcriptional response to microbial infection was examined. Systemic and oral infections were established with injecting or feeding Gram-positive bacteria *Staphylococcus aureus* and Gram-negative bacteria *Escherichia coli* DH5α as described by Dawadi et al. (2018). *Bacillus thuringiensis* is a biological control agent against RPW larvae (Pu et al., 2017). *Beauveria bassiana* was also used as fungal challenge and cultured on potato dextrose agar (PDA) for 2 weeks at 25°C. The conidial concentration of *B. bassiana* (1 × 10^8^ spores/ml) was determined with a Neubauer hemocytometer as described by Hussain et al. (2016). Fat bodies and guts of the microbe-challenged larvae were obtained at different time points (6, 12, and 24 h). To detect the transcript changes of *RfSpätzle* by microbial challenge, RT-qPCR was performed as described above.

### Functional Analysis of *RfSpätzle* by RNAi

To investigate the function of *RfSpätzle* in RPW immunity, the RNAi technique was used to silence this target gene. Following the manufacturer’s protocols, the MEGA script*^®^* RNAi Kit (Thermo Fisher Scientific, United States) was used to synthesize *RfSpätzle* and eGFP dsRNA. In this study, eGFP dsRNA was employed as the control for our RNAi experiments. dsRNA (1 μg) was injected into the body cavity of fourth instar larvae. According to our previous investigations (Dawadi et al., 2018; Xiao et al., 2019), fat bodies, and guts were dissected 48 h after dsRNA delivery to verify RNAi efficiency by RT-qPCR.

### Effect of *RfSpätzle* Knockdown on the Survival Ability of RPW Larvae

To evaluate the effect of *RfSpätzle* knockdown on the survival rate of RPW larvae, 48 h after the delivery of dsRNA, *RfSpätzle*-silenced individuals were challenged with entomopathogenic bacteria, *B. thuringiensis* strain HA (Pu et al., 2017). Five microliters of bacterial suspension (1.0 × 10^8^ CFU/ml) or the same volume of sterile PBS (as control) was injected into the hemocoel of the fourth instar larvae (*n* = 30) and the survival rate was monitored every 6 h. The effect of *RfSpätzle* knockdown on the survival rate was determined with the Kaplan–Meier Log Rank survival test (IBM SPSS Statistics 22.0).

### Effect of *RfSpätzle* Knockdown on the Gut Bacterial Composition of RPW

To reveal the impact of *RfSpätzle* knockdown on gut bacterial load and composition, guts were aseptically pulled out from RPW larvae at 48 h after dsRNA delivery and homogenized. Three larvae were dissected as a replicate and each treatment comprised at least three replicates. After serial dilution, gut homogenate was spread on LB agar plates and incubated for 16 h at 37°C as described by Dawadi et al. (2018). The number of colony-forming units (CFUs) of gut bacteria from the treated insects was counted. Furthermore, gut homogenate was processed for extracting total bacterial genomic DNA as described by Muhammad et al. (2017). Additionally, a blank DNA extraction was conducted as explained above but without any gut samples in each replicate. The bacterial 16S rRNA hypervariable region (V3–V4) was amplified with gene-specific primers 338F 5′-ACTCCTACGGGAGGCAGCAG-3′ and 5′-GGACTACHVGGGTWTCTAAT-3′. In the negative control, an equal volume of sterilized ddH_2_O was used as the template to check for laboratory contamination. No target PCR products were detected by 1% agarose gel electrophoresis in any negative controls. The sequencing was completed with the Majorbio I-Sanger (China) Illumina MiSeq platform. The OTUs (operational taxonomic units) were clustered at the similarity threshold of 97% with the Usearch program (version 7.0^5^). Taxonomic analysis was performed with RDP Classifier^6^ against the Bacteria database (Silva: Release128^7^ and Greengene: Release 13.5^8^) at the confidence threshold of 0.7. The sequencing data were processed and analyzed with the methods as described by Muhammad et al. (2017) and Dawadi et al. (2018). Briefly, beta diversity analysis of RPW gut bacteria was completed through principal coordinate analysis (PCoA) and principal component analysis (PCA). ANOSIM (analysis of similarity) and DEseq analysis were employed to detect the differences in the gut bacterial community between the two groups.

## Results

### Molecular Characterization of *RfSpätzle*

**FIGURE 1:**
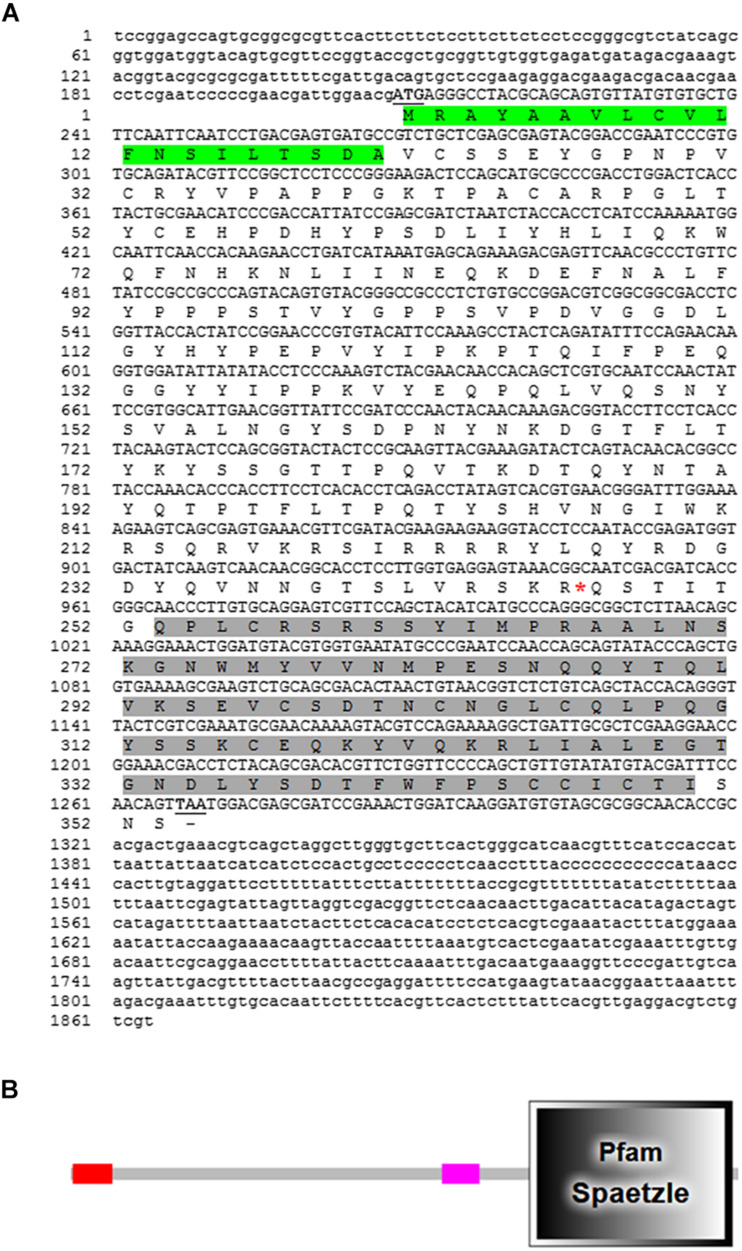
Full-length cDNA nucleotide and deduced amino acid sequence of *RfSpätzle* **(A)** and schematic representation of the conserved functional domains in its deduced polypeptide sequence **(B)**. The start and stop codons are underlined and shown in bold letters. The signal peptide is shaded in green, and the conserved domain is dark gray. In the schematic representation, the signal peptide and Spätzle (Spz) domain are shown with the red and dark gray rectangles, respectively.

**FIGURE 2:**
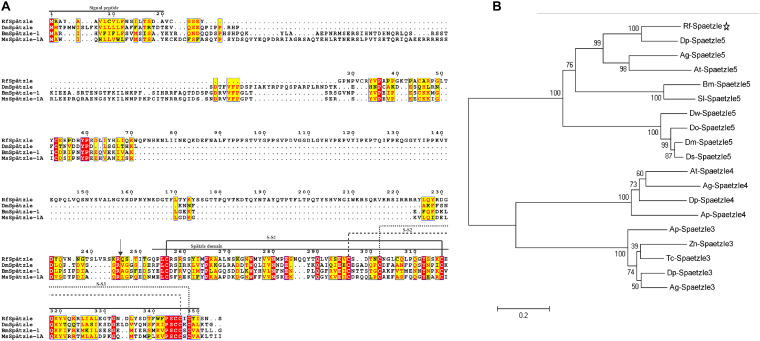
Multiple alignments **(A)** and phylogenetic analysis **(B)** of *Rf*Spätzle with other **Spz** proteins. The arrow indicates the predicted cleavage site in RfSpätzle. The maximum likelihood (ML) method of MEGA 5.05 was employed to construct the phylogenetic tree of Spz sequences with 5000 bootstrap replicates. The followin*g* Spz were retrieved for our phylogenetic analysis: Bm-Spaetzle 5 [*Bombyx mori* AMR08002.1], Sl-Spaetzle 5 [*Spodoptera litura* XP_022816314.1], Zn-Spaetzle 3 [*Zootermopsis nevadensis* XP_021922319.1], Dm-Spaetzle 5 [*Drosophila melanogaster* NP_647753.1], Ds-Spaetzle 5 [*Drosophila serrata* XP_020809778.1], Do-Spaetzle 5 [*Drosophila obscura* XP_022208379.1], Dw-Spaetzle 5 [*Drosophila willistoni* XP_002062599.1], Dp-Spaetzle 3 [*Dendroctonus ponderosae* XP_019758543.1], Ag-Spaetzle 3 [*Anoplophora glabripennis* XP_018561823.2], Tc-Spaetzle 3 [*Tribolium castaneum* NP_001153625.1], Ap-Spaetzle 3 [*Agrilus planipennis* XP_025831835.1], Dp-Spaetzle 4 [*Dendroctonus ponderosae* XP_019771425.1], At-Spaetzle 4 [*Aethina tumida* XP_019880911.1], Ap-Spaetzle 4 [*Agrilus planipennis* XP_018325475.1], Ag-Spaetzle 4 [*Anoplophora glabripennis* XP_018575280.1], Dp spaetzle 5 [*Dendroctonus ponderosae* XP_019764273.1], Ag-Spaetzle 5 [*Anoplophora glabripennis* XP_018568892.1], and At-Spaetzle 5 [*Aethina tumida* XP_019879590.1].

The full-length cDNA sequence of *RfSpätzle* is 1865 bp long with a 1270-bp ORF that encodes a putative protein of 353 amino acids (Figure 1A), comprising an N-terminal signal peptide and a Spz domain of 97 amino acids at the C-terminus (Figure 1B). The presence of the Spz domain indicated that RfSpätzle is a member of the Spz family, mediating the Toll signaling pathway by binding the Toll receptors. The calculated mass and isoelectric point of this mature proSpätzle are 40,122.18 Da and 8.81. The putative activation cleavage site is located after VRSKR246 in pro-RfSpätzle, implying that pro-RfSpätzle might be activated by a clip-domain serine proteinase with trypsin-like specificity via limited proteolysis immediately after Arg^246^ (Jang et al., 2006; Wang et al., 2007; An et al., 2010). Interestingly, some residues, including six cysteines, two prolines, and one glutamine, are conserved in four insect species (Figure 2A). It has been confirmed that these six conserved cysteines are crucial to disulfide formation and fold stability of the neurotrophin-like cysteine knot (CK) structural motif (Jang et al., 2006; An et al., 2010). Moreover, most Spz members have an orphan cysteine before Cys5, which can establish an intermolecular linkage with its counterpart in another subunit to form a disulfide-linked homodimer (Hoffmann et al., 2008; Wang and Zhu, 2009; An et al., 2010). Furthermore, when 19 Spz sequences were taken into consideration for phylogenetic analysis, these sequences were split into three distinct clusters (Spz3, Spz4, and Spz5, Figure 2B). The inclusion of *RfSpätzle* in the same branch as *Drosophila* Spz-5, exhibiting a bootstrap value of 100, indicates that *RfSpätzle* is an ortholog of *Drosophila* Spz-5.

### Expression Profile of *RfSpätzle* Across Different Tissues and Its Response to the Challenge of Pathogenic Microbes

**FIGURE 3:**
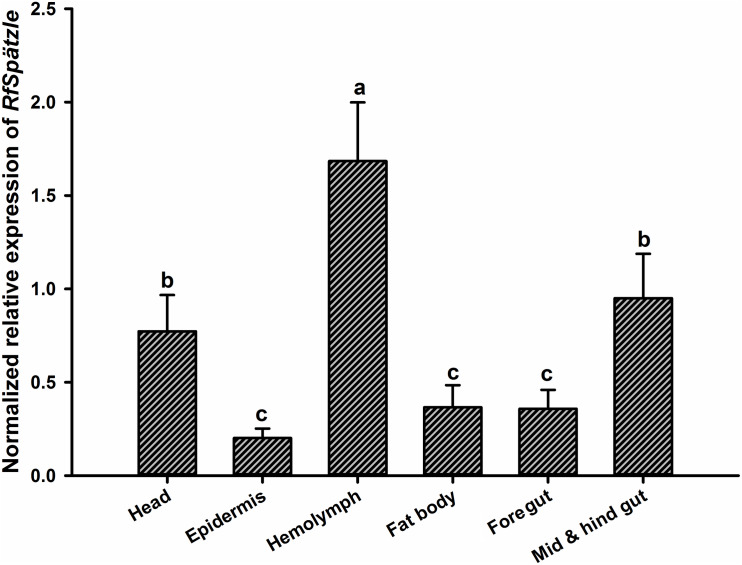
Expression level of *RfSpätzle* in various tissues of healthy RPW larvae was quantified by RT-qPCR. The expression level of *RfSpätzle* was normalized to the *Rf*β*-actin* gene. Different letters represent the significance, which was determined by Tukey’s HSD (Honest Significant Difference) test at *P* < 0.05. The data are shown as the mean ± SD of four independent replicates.

**FIGURE 4:**
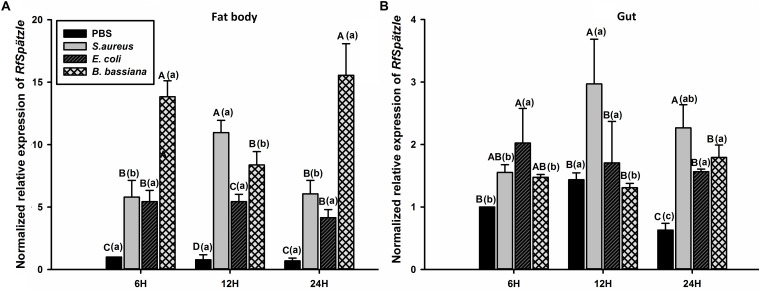
Transcriptional response of *RfSpätzle* in fat body **(A)** and gut **(B)** of RPW larvae after the systemic challenge of microbial pathogens at different time points. The expression level of *RfSpätzle* was normalized to the *Rf*β*-actin* gene. Uppercase letters indicate significant differences across all pathogen-challenged samples at the same point, while the lowercase letters represent the significance of the same treatment across different time points. The significance was determined by Tukey’s HSD test at *P* < 0.05. The data are shown as the mean ± SD of three independent replicates.

**FIGURE 5:**
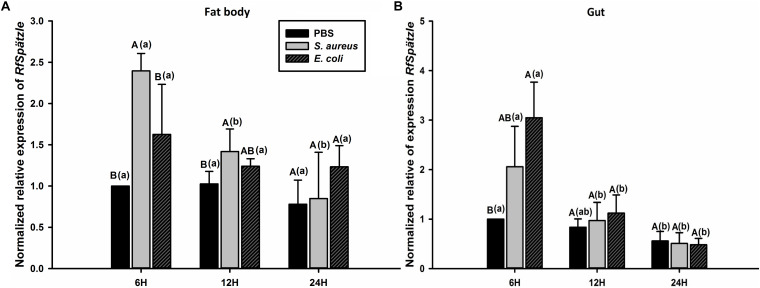
Transcriptional response of *RfSpätzle* in fat body **(A)** and guts **(B)** of RPW larvae at different time points after being orally infected by *S. aureus* and *E. coli*. The expression level of *RfSpätzle* was normalized to the *Rf*β*-actin* gene. Uppercase letters indicate significant differences across the different challenged samples at the same time point, while the lowercase letters represent the significance of the same treatment across different time points. The significance was detected by Tukey’s HSD test at *P* < 0.05. The data are shown as the mean ± SD of three independent replicates.

RT-qPCR analysis showed that *RfSpätzle* was constitutively expressed in the tested tissues at significantly different levels (ANOVA: *F*_5_,_18_ = 45.11, *P* < 0.001) (Figure 3). The expression level of *RfSpätzle* in the hemolymph and gut was 8.38- and 5.00-fold higher than that in the epidermis. *RfSpätzle* was significantly induced in the fat body and gut upon the systemic and oral pathogenic challenge of *S. aureus*, *E. coli*, and *B. bassiana* as compared to the controls (Figures 4, 5). These results suggest that *RfSpätzle* might mediate the systemic and gut immune responses to fight against microbial intruders.

### *RfSpätzle* Knockdown Impaired the Immune Response Against Pathogen Invasion

**FIGURE 6:**
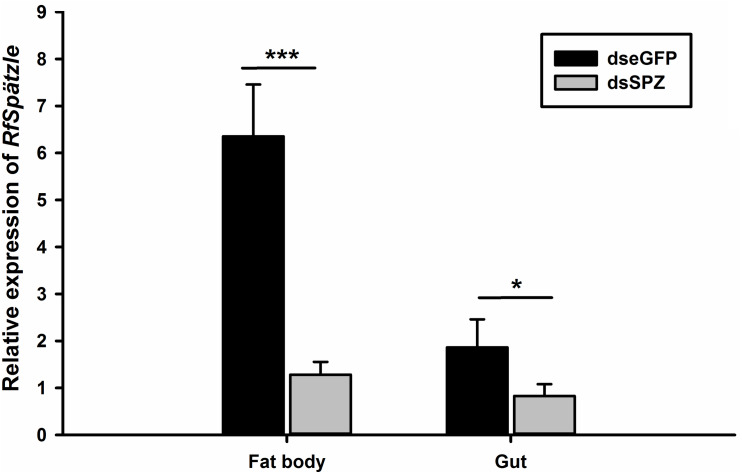
Verification of the *RfSpätzle* RNAi efficiency in the fat body and gut of the fourth instar RPW larvae 48 h after dsRNA injection. The data are shown as the mean ± SD of four independent replicates. The significant differences were determined by an independent sample *t* test (*P < 0.05 and ***P < 0.001).

**FIGURE 7:**
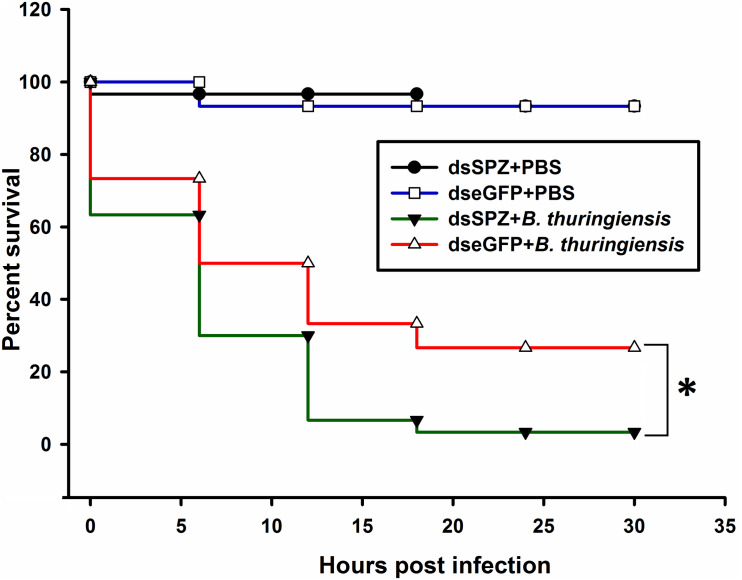
Survival rate of *RfSpätzle*-silenced individuals after being challenged by the pathogenic bacteria *Bacillus thuringiensis* strain HA. Survival analysis was performed with Kaplan-Meir survival log rank (Mantel-Cox) test (*P < 0.05). The experiment was repeated three times with 30 fourth instar larvae from each cohort.

**FIGURE 8:**
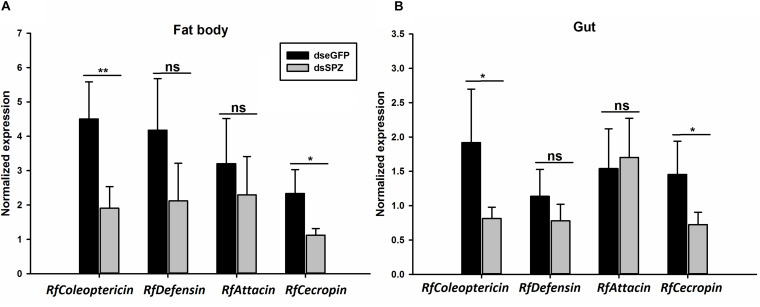
Effects of *RfSpätzle* knockdown on the transcript abundance of four antimicrobial peptide genes, *RfColeoptericin*, *RfDefensin*, *RfAttacin*, and *RfCecropin* in the fat body **(A)** and gut **(B)** of the fourth instar RPW larvae 48 h after dsRNA delivery. The data are shown as the mean ± SD of four independent replicates. The significant differences were detected with an independent sample *t* test (ns, non-significant, *P* > 0.05, *P < 0.05, and **P < 0.01).

Compared to the controls, the expression levels of *RfSpätzle* in the fat body and gut were significantly reduced by 83.23 and 69.26% at 48 h after dsRNA injection (*t* test for fat body: *t* = −8.95, df = 6, *P* < 0.001; gut: *t* = −2.92, df = 6, *P* < 0.05) (Figure 6). After being challenged by *B. thuringiensis*, *Rf*Spätzle-silenced individuals (dsSPZ) succumbed significantly faster than their counterparts (*P* < 0.05, Figure 7). Further investigations revealed that *RfSpätzle* silencing led to the significant downregulation of *RfColeoptericin* (*t* test for fat body: *t* = −4.18, df = 6, *P* = 0.006; gut: *t* = −2.77, df = 6, *P* < 0.05) and *RfCecropin* (*t* test for fat body: *t* = −3.34, df = 6, *P* < 0.05; gut: *t* = −2.83, df = 6, *P* < 0.05) in fat body and gut, but the expression level of *RfDefensin* (*t* test for fat body: *t* = −8.95, df = 6, *P* = 0.06; gut: *t* = −1.55, df = 6, *P* = 0.17) and *RfAttacin* (*t* test for fat body: *t* = −1.05, df = 6, *P* = 0.33; gut: *t* = −2.92, df = 6, *P* = 0.70) was not affected by *RfSpätzle* knockdown (Figures 8A,B). These findings suggested that *RfSpätzle* mediates the expression of these two antimicrobial peptide genes (*RfColeoptericin* and *RfCecropin*) to confer RPW protection against the pathogen invasion.

### *RfSpätzle* Knockdown Altered the Community Structure of RPW Gut Microbiota

**FIGURE 9:**
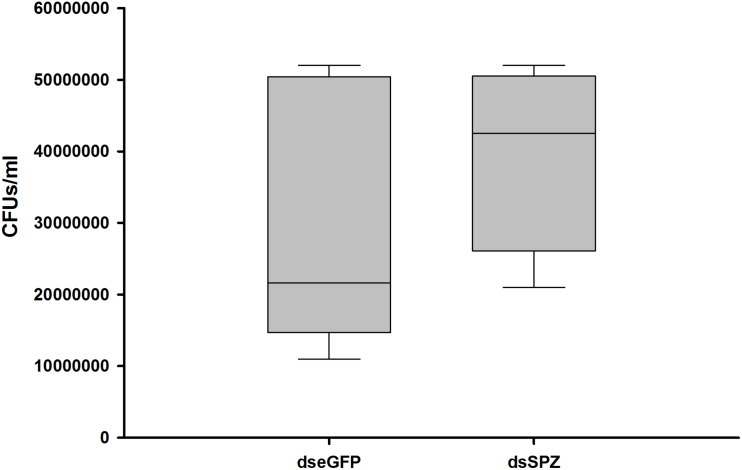
Impact of *RfSpätzle* silencing on the gut bacterial load of RPW larvae.

**FIGURE 10:**
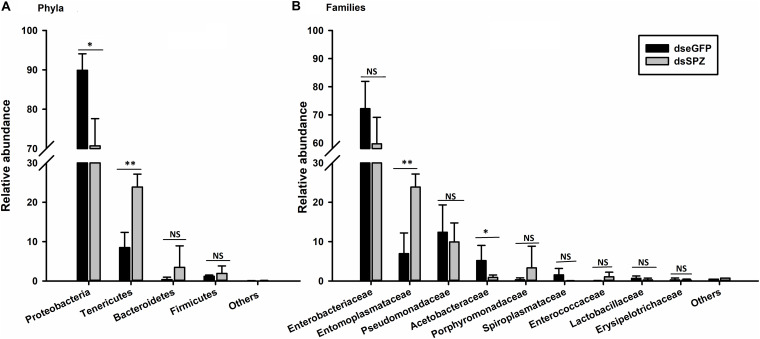
Influence of *RfSpätzle* silencing on the relative abundance of gut bacteria at the phylum **(A)** and family **(B)** levels. The data are shown as the mean ± SD of three independent replicates. The significant differences were detected with an independent sample *t* test (ns, non-significant, *P* > 0.05, *P < 0.05, and **P < 0.01).

The role of the *RfSpätzle-*mediated Toll-like pathway in regulating the composition and proportion of RPW gut bacteria was determined. Comparative analysis of the gut bacterial load showed a numerically higher number of CFUs in the *RfSpätzle-*silenced individuals although no significance was detected (*t* test: *t* = −1.22, df = 16, *P* = 0.24) (Figure 9). Bacterial 16S rRNA-based high-throughput sequencing yielded 431,163 reads. At the 97% similarity threshold, these reads were binned into 414 OTUs, which contained 243 OTUs from the dseGFP group and 171 OTUs from the dsSPZ group. Seventy OTUs were shared between the two groups (Supplementary Figure S1). Diversity analysis revealed that the community diversity (Shannon and Simpson) and species richness (ACE, Chao, and Sobs) did not vary significantly between the two groups (Table 2 and Supplementary Figure S2). Proteobacteria and Tenericutes represented the bulk (95%) of the RPW gut microbiota, while the relative abundance of Bacteroidetes and Firmicutes was less than 5%. At the family level, the microbiota was dominated (>98%) by Enterobacteriaceae, Entomoplasmataceae, Pseudomonadaceae, Acetobacteraceae, Porphyromonadaceae, Spiroplasmataceae, and Enterococcaceae. ANOSIM analysis revealed that no significance was determined in the RPW gut bacterial community between the two groups (*P* = 0.75). However, the relative abundance of gut bacteria at different taxonomic levels was altered by *Rf*Spätzle silencing. For example, the percentage of Proteobacteria (*t* test: *t* = 4.12, df = 4, *P* < 0.05) in *RfSpätzle*-silenced RPW larvae was decreased by 20% compared to that of controls, while the relative abundance of Tenericutes (*t* test: *t* = −5.27, df = 4, *P* < 0.05) was increased to 23.86% by *RfSpätzle* knockdown (Figure 10A). Similarly, the relative abundance of Acetobacteraceae (*t* test: *t* = 3.84, df = 4, *P* < 0.05) was significantly less than that of controls. However, Entomoplasmataceae (*t* test: *t* = −4.71, df = 4, *P* < 0.05) represented a higher percentage of *Rf*Spätzle-silenced RPW larvae (Figure 10B). At the OTU level, DEseq analysis revealed that the abundance of OTU306 (Cellulomonadaceae, G^+^), OTU279 (Gallionellaceae, G^–^), and OTU283 (*Ferriphaselus*, G^–^) was significantly increased while that of OTU200 (*Acetobacterium*, G^+^) was decreased by *RfSpätzle* silencing (Supplementary Table S1). Collectively, these data indicated that *RfSpätzle* knockdown can cause some significant changes in the proportion of RPW gut bacteria, suggesting that the Spz -mediated Toll-like pathway is involved in modulating the homeostasis of the RPW gut microbiota.

**TABLE 2:** Community diversity and richness of microbiota associated with the guts of control (dseGFP) and Spz knockdown (dsSPZ) RPW individuals.

		Community diversity		Community richness		
			
Sample ID	Reads	Shannon	Simpson	ACE	Chao	Sobs
dseGFP-1	52,972	1.4	0.39	55.1	53.0	49
dseGFP-2	50,724	0.8	0.68	168.1	66.5	41
dseGFP-3	51,848	1.1	0.52	43.7	40.1	38
dsSPZ-1	57,258	1.0	0.47	71.5	50.8	41
dsSPZ-2	57,296	1.1	0.51	81.5	76.1	72
dsSPZ-3	51,553	1.2	0.38	40.0	37.0	34

## Discussion

Recently, the immune responses of RPW larvae to some biocontrol agents, including the entomopathogenic nematode *Steinernema carpocapsae* (Mastore et al., 2014), bacteria (Shi et al., 2014), and fungi (Hussain et al., 2016), have been investigated. However, the underlying molecular mechanisms conferring RPW larvae to ward off the microbial pathogens have not been fully elucidated. In this work, a homolog of *D*. *melanogaster* Spz, *RfSpätzle*, was first cloned and characterized from *R*. *ferrugineus*. *RfSpätzle* encodes a protein that consists of two typical conserved functional domains, a signal peptide and a Spz domain. Multiple sequence comparisons showed that a putative activation cleavage site of *RfSpätzle* is directly after R^246^, suggesting that an activating proteinase could cleave pro-*RfSpätzle* after this specific Arg to release the activated form of *RfSpätzle*. Interestingly, seven Cys residues, which are found in nearly all known Spz cysteine-knot domains, were also found in the C-terminal active cystine-knot domain of *Rf*Spätzle. These data suggested that *Rf*Spätzle is a secretory cysteine-knot protein. Moreover, the conserved features of this protein implied that the *RfSpätzle* homodimer can be formed via intermolecular Cys-Cys disulfide bonds to activate the immune signaling pathway (Ferrandon et al., 2007; An et al., 2010; Zhong et al., 2012; Vaniksampanna et al., 2019).

*RfSpätzle* is constitutively expressed at different levels in all tested tissues of RPW larvae. The expression level of *RfSpätzle* was significantly higher in gut and hemolymph compared to other tissues, indicating that it might be involved in the systemic and gut immunity of RPW larvae. Moreover, the challenge of *S. aureus*, *E. coli*, and *B. bassiana* strongly induced the expression of *RfSpätzle* in the fat body and gut of RPW larvae. Additionally, we found that *RfSpätzle-*silenced individuals were more vulnerable to pathogenic infection than controls. Further analysis confirmed that *RfSpätzle* silencing resulted in the significant downregulation of *RfColeoptericin* and *RfCecropin*, suggesting that *RfSpätzle* knockdown could compromise RPW innate immunity. Interestingly, the secretion of cecropin is IMD-dependent in *D. melanogaster* (Önfelt Tingvall et al., 2001), and coleoptericins have been shown to be IMD-dependent in the cereal weevil *Sitophilus* (Maire et al., 2018). Recently, a similar role of the Spz gene was also revealed in shrimp *Litopenaeus vannamei* (Yuan et al., 2017), the black tiger shrimp *P. monodon* (Boonrawd et al., 2017), and freshwater prawn *M. rosenbergii* (Vaniksampanna et al., 2019). It is known that *D. melanogaster* Spz, an extracellular ligand of the Toll receptor, is required to activate the production of antimicrobial peptides via the Toll pathway (Valanne et al., 2011). Collectively, these data indicate that RfSpätzle can mediate the secretion of some antimicrobial peptides of RPW larvae to defend against microbial intruders.

The gut microbiota has been confirmed to profoundly affect host physiological fitness, including development, nutrition metabolism, and immunity, in many metazoan animals (Muhammad et al., 2017; Zheng et al., 2017; Kamareddine et al., 2018; Hebineza et al., 2019). Importantly, the healthy homeostasis of gut microbiota is critical for proper growth and development of the host. However, the exact mechanisms by which animals tolerate the commensal bacteria while eliminating the transient pathogenic bacteria are still not well understood. In *D. melanogaster*, it is well-known that dual oxidase-mediated ROS production and the IMD pathway play vital roles in regulating the homeostasis of gut microbiota (Ryu et al., 2008; Lee et al., 2017). More recently, the *RfRelish*-mediated IMD-like pathway has been determined to be involved in modulating the homeostasis of RPW gut bacteria (Dawadi et al., 2018; Xiao et al., 2019). Insect immune responses are mainly under the control of two signaling pathways, the Toll and IMD pathways (Hetru and Hoffmann, 2009). However, the Toll pathway does not mediate local gut immunity in *D. melanogaster* (Davis and Engström, 2012). Previously, our transcriptome analysis found the essential elements for the Toll signaling pathway, including SPs, Spz-like proteins, Toll receptors, Tube-1, MyD88, TRAF, and Cactus, in the RPW gut (Muhammad et al., 2019). In the present investigation, we found that the expression levels of *RfSpätzle* and some antimicrobial peptides were drastically induced in the fat body and gut upon microbial infection. Specifically, it was found that the relative abundance of some gut bacteria in RPW larvae was significantly altered by *RfSpätzle* knockdown. The expression levels of antimicrobial peptides in the gut, *RfColeoptericin* and *RfCecropin*, were also reduced by *RfSpätzle* silencing. Antimicrobial peptides are one of the vital effectors to maintain insect-microbe symbiosis (Ryu et al., 2008; Login et al., 2011; Vigneron et al., 2012; Masson et al., 2015; Lee et al., 2017; Dawadi et al., 2018; Zaidman-Rémy et al., 2018; Xiao et al., 2019). Consequently, our data suggested that the *RfSpätzle*-mediated Toll-like signaling pathway could regulate the proportion of RPW gut bacteria by mediating the synthesis of antimicrobial peptides. To the best of our knowledge, this report is the first to reveal the possible role of the Spz-mediated Toll-like pathway in regulating the homeostasis of insect gut microbiota. However, *RfSpätzle* knockdown only differentially affected the relative abundance of four OTUs, including gram-positive and gram-negative bacteria, suggesting that the effects of the Toll-like signaling pathway on gut bacterial composition are highly limited and non-specific. It has been revealed that the majority of RPW gut bacteria are Gram-negative (Muhammad et al., 2017). This finding might explain the dominant role of the IMD-like pathway in maintaining the homeostasis of RPW gut bacteria.

It has been well defined that many weevils are associated with the γ-proteobacterial endosymbiont lineage *Nardonella*, which produces tyrosine for host cuticle formation and hardening (Hosokawa et al., 2015; Anbutsu et al., 2017). Although the weevil–*Nardonella* coevolution has lasted more than 100 million years, *Nardonella* infections in a number of weevil lineages have been lost or replaced by different bacterial lineages (Lefèvre et al., 2004; Conord et al., 2008). Therefore, although we still did not find *Nardonella* in RPW populations of our country in this study, it would be worthwhile to intensively investigate the presence of *Nardonella* in other beetle species to uncover the strikingly dynamic aspect of the endosymbiotic evolution in insects. The topic of insect immunity preserving endosymbionts and controlling their load and location while keeping the ability to cope with potential environmental infections by microbial intruders is highly interesting. Recently, the IMD-like pathway has been confirmed to mediate the control of *Sodalis pierantonius* in the bacteriome of *Sitophilus* spp. (Maire et al., 2018). However, whether the Toll pathway is involved in modulating the endosymbionts in bacteriome has not been determined. Moreover, the IMD and Toll pathways in hemipterans and other arthropods might be intertwined to target wider and overlapping arrays of microbes (Nishide et al., 2019). Therefore, further studies can detect whether there is any crosstalk between the IMD and Toll signaling pathways in this pest to regulate the homeostasis of RPW gut bacteria.

## Conclusion

In conclusion, we determined that *RfSpätzle* is involved in the innate immunity of RPW by mediating the secretion of the antimicrobial peptides *RfColeoptericin* and *RfCecropin*. The *Rf*Spätzle-mediated Toll-like signaling pathway not only confers protection against pathogen invasion but may also modulate the proportion of some gut bacteria.

## Data Availability Statement

The datasets generated for this study can be found in the NCBI database and the submission number of our sequence data is SAMN11959955-11959960.

## Author Contributions

ZS conceived and designed the research. ZS and YH provided the reagents. AM, PH, RX, and TJ completed the experiments. AM, XW, and ZS analyzed the data. ZS and AM prepared the manuscript. All authors have read and approved the final manuscript.

## Conflict of Interest

The authors declare that the research was conducted in the absence of any commercial or financial relationships that could be construed as a potential conflict of interest.

## Footnotes

1 http://www.expasy.org/

2 http://www.smart.embl-heidelberg

3 https://blast.ncbi.nlm.nih.gov/Blast.cgi

4 https://www.ebi.ac.uk/Tools/msa/clustalo/

5 http://drive5.com/uparse/

6 http://rdp.cme.msu.edu/

7 http://www.arb-silva.de

8 http://greengenes.secondgenome.com/

## Supplementary Material

FIGURE S1 Bacterial OTUs (operational taxonomic units) were recovered from the control insects (dseGFP) and *RfSpätzle*-silenced individuals (dsSPZ). Venn diagram indicates the unique and shared OTUs between the two groups.

FIGURE S2 Principal coordinate analysis of the phylogenetic β-diversity matrix obtained starting from the OTU table. The explained variance is as follows: 62.71% 1st component, 27.53% 2nd component.

TABLE S1 Effect of *R*f*Spätzle* knockdown on the reads number of OTUs between the two groups.
