# Supplementary figures and images for: Spätzle Homolog-Mediated Toll-Like Pathway Regulates Innate Immune Responses to Maintain the Homeostasis of Gut Microbiota in the Red Palm Weevil, Rhynchophorus ferrugineus Olivier (Coleoptera: Dryophthoridae)
Source: Front Microbiol. 2020 May 25;11:846. doi: 10.3389/fmicb.2020.00846 (PMC7261851; doi:10.3389/fmicb.2020.00846)

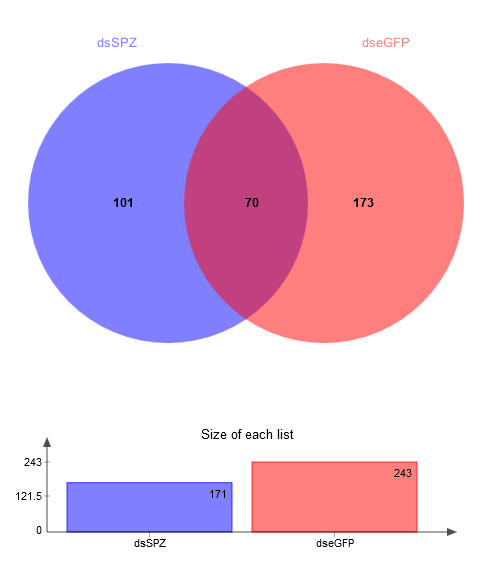

Supplement: FIGURE S1 — Bacterial OTUs (operational taxonomic units) were recovered from the control insects (dseGFP) and RfSpätzle-silenced individuals (dsSPZ). Venn diagram indicates the unique and shared OTUs between the two groups. [file Image_1.png]

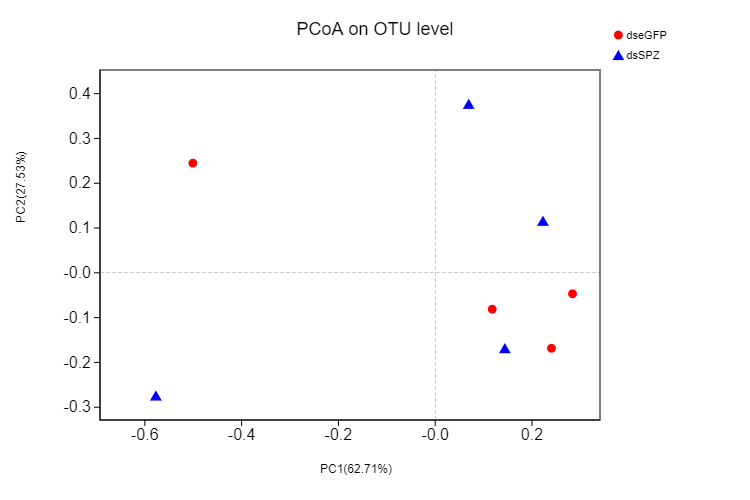

Supplement: FIGURE S2 — Principal coordinate analysis of the phylogenetic β-diversity matrix obtained starting from the OTU table. The explained variance is as follows: 62.71% 1st component, 27.53% 2nd component. [file Image_2.TIF]
