# Supplementary material for: Spätzle Homolog-Mediated Toll-Like Pathway Regulates Innate Immune Responses to Maintain the Homeostasis of Gut Microbiota in the Red Palm Weevil, Rhynchophorus ferrugineus Olivier (Coleoptera: Dryophthoridae)
Source: Front Microbiol. 2020 May 25;11:846. doi: 10.3389/fmicb.2020.00846 (PMC7261851; doi:10.3389/fmicb.2020.00846)
Supplement: TABLE S1 — Effect of RfSpätzle knockdown on the reads number of OTUs between the two groups. [file Table_1.DOCX]

**Table S1. Effect of *R*f*Spätzle* knockdown on the reads number of OTUs between the two groups.**

| **OTUs** | **Families** | **Genera** | **Reads count in dseGFP** | **Reads count in dsSPZ** | ***P*-value** | |
| --- | --- | --- | --- | --- | --- | --- |
| OTU279 | Gallionellaceae | unclassified | 1 | 12 | 0.04 |  |
| OTU200 | Eubacteriaceae | *Acetobacterium* | 465 | 5 | 0.04 |  |
| OTU283 | Gallionellaceae | *Ferriphaselus* | 2 | 24 | 0.04 |  |
| OTU306 | Cellulomonadaceae | unclassified | 2 | 17 | 0.04 |  |
| OTU186 | Chitinophagaceae | *Taibaiella* | 150 | 17 | 0.05 |  |
| OTU226 | Oxalobacteraceae | unclassified | 339 | 705 | 0.07 |  |
| OTU141 | Geobacteraceae | *Geobacter* | 166 | 45 | 0.07 |  |
| OTU179 | Acidiferrobacteraceae | *Sulfurifustis* | 8 | 0 | 0.07 |  |
| OTU340 | Hydrogenimonadaceae | *Sulfurospirillum* | 0 | 14 | 0.07 |  |
| OTU331 | Methylobacteriaceae | *Methylobacterium* | 1 | 4 | 0.07 |  |
| OTU137 | Sphingomonadaceae | *Sphingomonas* | 131 | 0 | 0.07 |  |
| OTU140 | Roseiflexaceae | *Roseiflexus* | 126 | 0 | 0.07 |  |
| OTU284 | Rhizobiaceae | *Candidatus* | 0 | 7 | 0.08 |  |
| OTU181 | Betaproteobacteria | unclassified | 4 | 0 | 0.08 |  |
| OTU134 | Prevotellaceae | unclassified | 96 | 0 | 0.08 |  |
| OTU299 | Bacteroidales | unclassified | 0 | 4 | 0.09 |  |
| OTU259 | Betaproteobacteria | unclassified | 0 | 14 | 0.09 |  |
| OTU80 | Micromonosporaceae | *Virgisporangium* | 73 | 0 | 0.09 |  |
| OTU280 | Sandaracinaceae | unclassified | 0 | 13 | 0.09 |  |
| OTU338 | Corynebacteriaceae | *Corynebacterium* | 0 | 73 | 0.09 |  |
| OTU276 | Clostridiales | unclassified | 0 | 4 | 0.10 |  |
| OTU324 | Dysgonamonadaceae | *Dysgonomonas* | 0 | 10 | 0.10 |  |
| OTU301 | Zoogloeaceae | *Thauera* | 0 | 10 | 0.10 |  |
| OTU345 | Morganellaceae | *Providencia* | 0 | 21 | 0.10 |  |
| OTU315 | Prevotellaceae | *Alloprevotella* | 4 | 19 | 0.10 |  |
| OTU105 | Cyanobacteria | unclassified | 59 | 0 | 0.10 |  |
| OTU12 | Acidobacteriaceae | unclassified | 59 | 0 | 0.10 |  |
| OTU341 | Helicobacteraceae | *Sulfuricurvum* | 0 | 26 | 0.11 |  |
| OTU277 | Acetobacteraceae | *Acidomonas* | 0 | 9 | 0.11 |  |
| OTU230 | Moraxellaceae | *Acinetobacter* | 0 | 5 | 0.11 |  |
| OTU36 | Eggerthellaceae | *Enterorhabdus* | 55 | 0 | 0.11 |  |
| OTU265 | Lactobacillaceae | *Lactobacillus* | 0 | 8 | 0.11 |  |
| OTU236 | Burkholderiaceae | *Ralstonia* | 0 | 8 | 0.11 |  |
| OTU89 | Chloroflexi | unclassified | 5 | 0 | 0.11 |  |
| OTU254 | Ignavibacteriales | unclassified | 0 | 7 | 0.12 |  |
| OTU287 | Propionibacteriaceae | *Propionicicella* | 0 | 7 | 0.12 |  |
| OTU318 | Clostridiaceae | *Clostridium* | 0 | 7 | 0.12 |  |
| OTU261 | Actinobacteria | *Gaiellales* | 0 | 7 | 0.12 |  |
| OTU253 | Erysipelotrichaceae | *Erysipelatoclostridium* | 0 | 7 | 0.12 |  |
| OTU56 | Peptostreptococcaceae | *Terrisporobacter* | 5 | 0 | 0.12 |  |
| OTU256 | Ruminococcaceae | *Ruminiclostridium* | 0 | 3 | 0.12 |  |
| OTU238 | Deltaproteobacteria | unclassified | 0 | 6 | 0.13 |  |
| OTU239 | Gallionellaceae | unclassified | 0 | 6 | 0.13 |  |
| OTU297 | Leptotrichiaceae | unclassified | 0 | 6 | 0.13 |  |
| OTU289 | Verrucomicrobiaceae | *Akkermansia* | 0 | 6 | 0.13 |  |
| OTU39 | Propionibacteriaceae | *Propionibacterium* | 38 | 0 | 0.13 |  |
| OTU55 | Rhodocyclaceae | *Denitratisoma* | 37 | 0 | 0.13 |  |
| OTU335 | Alcaligenaceae | *Achromobacter* | 0 | 13 | 0.13 |  |
| OTU202 | Helicobacteraceae | *Helicobacter* | 4 | 0 | 0.14 |  |
| OTU148 | Ruminococcaceae | unclassified | 32 | 0 | 0.14 |  |
| OTU269 | Comamonadaceae | *Polaromonas* | 0 | 5 | 0.14 |  |
| OTU300 | Gallionellaceae | *Candidatus* | 0 | 5 | 0.14 |  |
| OTU319 | Prevotellaceae | *Prevotella* | 0 | 5 | 0.14 |  |
| OTU326 | Proteobacteria | unclassified | 0 | 5 | 0.14 |  |
| OTU292 | Clostridiaceae | unclassified | 0 | 5 | 0.14 |  |
| OTU260 | Veillonellaceae | *Megamonas* | 0 | 5 | 0.14 |  |
| OTU290 | Clostridia | unclassified | 0 | 5 | 0.14 |  |
| OTU320 | Chitinophagaceae | *Terrimonas* | 0 | 5 | 0.14 |  |
| OTU270 | Oxalobacteraceae | *Undibacterium* | 0 | 5 | 0.14 |  |
| OTU264 | Chitinophagaceae | unclassified | 9 | 11 | 0.15 |  |
| OTU332 | Flavobacteriaceae | *Chryseobacterium* | 0 | 3 | 0.15 |  |
| OTU235 | Intrasporangiaceae | unclassified | 0 | 4 | 0.16 |  |
| OTU245 | Burkholderiaceae | *Pandoraea* | 0 | 4 | 0.16 |  |
| OTU266 | Comamonadaceae | *Burkholderia* | 0 | 4 | 0.16 |  |
| OTU240 | Erysipelotrichaceae | *Allobaculum* | 0 | 4 | 0.16 |  |
| OTU234 | Flavobacteriaceae | *Cloacibacterium* | 0 | 4 | 0.16 |  |
| OTU248 | Halomonadaceae | *Halomonas* | 0 | 4 | 0.16 |  |
| OTU308 | Ruminococcaceae | unclassified | 0 | 4 | 0.16 |  |
| OTU316 | Ruminococcaceae | unclassified | 0 | 4 | 0.16 |  |
| OTU322 | Bacteroidales | unclassified | 0 | 4 | 0.16 |  |
| OTU325 | Syntrophomonadaceae | unclassified | 0 | 4 | 0.16 |  |
| OTU307 | Prevotellaceae | unclassified | 0 | 4 | 0.16 |  |
| OTU76 | Bacteriovoracaceae | *Bacteriovorax* | 24 | 0 | 0.16 |  |
| OTU45 | Hyphomicrobiaceae | *Devosia* | 5 | 6 | 0.16 |  |
| OTU32 | Rhodocyclaceae | *Dechloromonas* | 23 | 0 | 0.17 |  |
| OTU4 | Sphingobacteriales | unclassified | 3 | 0 | 0.17 |  |
| OTU334 | Thermomicrobia | unclassified | 21 | 40 | 0.17 |  |
| OTU69 | Comamonadaceae | *Rhodoferax* | 22 | 0 | 0.17 |  |
| OTU185 | Porphyromonadaceae | *Parabacteroides* | 3 | 0 | 0.18 |  |
| OTU190 | Rhodospirillales | unclassified | 3 | 0 | 0.18 |  |
| OTU204 | Betaproteobacteria | unclassified | 0 | 4 | 0.19 |  |
| OTU205 | Rhodocyclaceae | *Sterolibacterium* | 0 | 4 | 0.19 |  |
| OTU223 | Streptococcaceae | *Lactococcus* | 0 | 4 | 0.19 |  |
| OTU59 | Prevotellaceae | *Prevotella* | 19 | 0 | 0.19 |  |
| OTU51 | Bacteroidetes | unclassified | 18 | 0 | 0.19 |  |
| OTU215 | Spirochaetaceae | *Sphaerochaeta* | 0 | 2 | 0.19 |  |
| OTU313 | Acidimicrobiales | unclassified | 1 | 4 | 0.19 |  |
| OTU271 | Prevotellaceae | unclassified | 0 | 3 | 0.19 |  |
| OTU323 | Comamonadaceae | *Acidovorax* | 0 | 3 | 0.19 |  |
| OTU242 | Leuconostocaceae | *Leuconostoc* | 0 | 3 | 0.19 |  |
| OTU250 | Flavobacteriaceae | *Elizabethkingia* | 0 | 3 | 0.19 |  |
| OTU273 | Acidobacteriaceae | *Edaphobacter* | 0 | 3 | 0.19 |  |
| OTU246 | Chthoniobacterales | unclassified | 0 | 3 | 0.19 |  |
| OTU247 | Peptococcaceae | *Thermincola* | 0 | 3 | 0.19 |  |
| OTU272 | Fusobacteriales | unclassified | 0 | 3 | 0.19 |  |
| OTU317 | Hyphomicrobiaceae | *Pedomicrobium* | 0 | 3 | 0.19 |  |
| OTU241 | Chromatiaceae | *Rheinheimera* | 0 | 3 | 0.19 |  |
| OTU312 | Micromonosporaceae | *Actinoplanes* | 0 | 3 | 0.19 |  |
| OTU201 | Peptostreptococcaceae | *Romboutsia* | 439 | 406 | 0.21 |  |
| OTU50 | Bifidobacteriaceae | *Bifidobacterium* | 15 | 0 | 0.21 |  |
| OTU210 | Berkelbacteria | unclassified | 0 | 3 | 0.22 |  |
| OTU206 | Rhodocyclaceae | *Sulfuritalea* | 0 | 3 | 0.22 |  |
| OTU228 | Oligoflexales | unclassified | 0 | 3 | 0.22 |  |
| OTU15 | Planococcaceae | *Solibacillus* | 14 | 0 | 0.22 |  |
| OTU310 | Bacteroidetes | unclassified | 10 | 17 | 0.22 |  |
| OTU197 | Dependentiae | unclassified | 2 | 0 | 0.23 |  |
| OTU199 | Coriobacteriaceae | unclassified | 2 | 0 | 0.23 |  |
| OTU136 | Bacillales | unclassified | 28 | 8 | 0.23 |  |
| OTU102 | Shewanellaceae | *Shewanella* | 13 | 0 | 0.23 |  |
| OTU95 | Acidothermaceae | *Acidothermus* | 13 | 0 | 0.23 |  |
| OTU38 | Prevotellaceae | unclassified | 13 | 0 | 0.23 |  |
| OTU3 | Enterobacteriaceae | unclassified | 2 | 0 | 0.23 |  |
| OTU173 | Peptostreptococcaceae | *Peptostreptococcus* | 2 | 0 | 0.23 |  |
| OTU184 | Ruminococcaceae | *Eubacterium* | 2 | 0 | 0.24 |  |
| OTU175 | Bacillaceae | *Fictibacillus* | 2 | 0 | 0.24 |  |
| OTU156 | Brocadiaceae | unclassified | 12 | 0 | 0.24 |  |
| OTU162 | Micromonosporaceae | *Micromonospora* | 12 | 0 | 0.24 |  |
| OTU174 | Saprospiraceae | unclassified | 7 | 1 | 0.24 |  |
| OTU333 | Syntrophorhabdaceae | *Syntrophorhabdus* | 0 | 3 | 0.24 |  |
| OTU344 | Comamonadaceae | *Curvibacter* | 5 | 13 | 0.25 |  |
| OTU183 | Ruminococcaceae | unclassified | 767 | 513 | 0.25 |  |
| OTU224 | Ruminococcaceae | *Ruminococcus* | 55 | 897 | 0.26 |  |
| OTU255 | Comamonadaceae | *Comamonas* | 0 | 2 | 0.26 |  |
| OTU275 | Rhodobacteraceae | *Paracoccus* | 0 | 2 | 0.26 |  |
| OTU293 | Lachnospiraceae | *Lachnoclostridium* | 0 | 2 | 0.26 |  |
| OTU314 | Oxalobacteraceae | *Duganella* | 0 | 2 | 0.26 |  |
| OTU257 | Iamiaceae | *Iamia* | 0 | 2 | 0.26 |  |
| OTU288 | Porphyromonadaceae | *Odoribacter* | 0 | 2 | 0.26 |  |
| OTU267 | Nitrospirae | *Nitrospira* | 0 | 2 | 0.26 |  |
| OTU274 | Desulfobulbaceae | *Desulfurivibrio* | 0 | 2 | 0.26 |  |
| OTU237 | Micrococcaceae | *Arthrobacter* | 0 | 2 | 0.26 |  |
| OTU263 | Candidatus | *Azambacteria* | 0 | 2 | 0.26 |  |
| OTU309 | Chloroflexi | unclassified | 0 | 2 | 0.26 |  |
| OTU249 | Micrococcaceae | *Pseudarthrobacter* | 0 | 2 | 0.26 |  |
| OTU278 | Gracilibacteria | unclassified | 0 | 2 | 0.26 |  |
| OTU233 | Helicobacteraceae | *Sulfurimonas* | 0 | 2 | 0.26 |  |
| OTU53 | Bacteroidaceae | *Bacteroides* | 10 | 0 | 0.27 |  |
| OTU111 | Spiroplasmataceae | *Spiroplasma* | 9 | 0 | 0.28 |  |
| OTU165 | Lachnospiraceae | *Ruminococcus* | 9 | 0 | 0.28 |  |
| OTU84 | Erysipelotrichaceae | *Erysipelothrix* | 9 | 0 | 0.28 |  |
| OTU75 | Rhodospirillaceae | *Defluviicoccus* | 9 | 0 | 0.28 |  |
| OTU209 | Peribacteria | unclassified | 0 | 2 | 0.28 |  |
| OTU212 | Xanthomonadaceae | *Lysobacter* | 0 | 2 | 0.28 |  |
| OTU207 | Acidobacteriaceae | unclassified | 0 | 2 | 0.28 |  |
| OTU208 | Chitinophagaceae | unclassified | 0 | 2 | 0.28 |  |
| OTU222 | Nitrosomonadales | unclassified | 0 | 2 | 0.28 |  |
| OTU219 | Omnitrophica | unclassified | 0 | 2 | 0.28 |  |
| OTU216 | Rhizobiales | unclassified | 0 | 2 | 0.28 |  |
| OTU220 | Comamonadaceae | *Limnohabitans* | 0 | 2 | 0.28 |  |
| OTU231 | Rhodospirillaceae | unclassified | 0 | 2 | 0.28 |  |
| OTU225 | Solirubrobacterales | unclassified | 0 | 2 | 0.28 |  |
| OTU221 | Acetobacteraceae | *Gluconobacter* | 0 | 2 | 0.28 |  |
| OTU188 | Solibacteraceae | *Solibacter* | 5 | 15 | 0.29 |  |
| OTU339 | Ignavibacteriales | unclassified | 0 | 2 | 0.29 |  |
| OTU268 | Alphaproteobacteria | unclassified | 1 | 7 | 0.30 |  |
| OTU116 | Rhizobiaceae | *Rhizobium* | 8 | 0 | 0.30 |  |
| OTU125 | Cyanobacteria | unclassified | 8 | 0 | 0.30 |  |
| OTU157 | Cyclobacteriaceae | *Algoriphagus* | 8 | 0 | 0.30 |  |
| OTU122 | Gaiellales | *Gaiella* | 8 | 0 | 0.30 |  |
| OTU6 | Microbacteriaceae | *Cryobacterium* | 8 | 0 | 0.30 |  |
| OTU91 | Cyclobacteriaceae | unclassified | 8 | 0 | 0.30 |  |
| OTU65 | Solirubrobacterales | unclassified | 8 | 0 | 0.30 |  |
| OTU40 | Rikenellaceae | unclassified | 8 | 0 | 0.30 |  |
| OTU327 | Lineage | unclassified | 0 | 2 | 0.31 |  |
| OTU329 | Burkholderiales | unclassified | 0 | 2 | 0.31 |  |
| OTU328 | Lachnospiraceae | unclassified | 0 | 2 | 0.31 |  |
| OTU330 | Gemmatimonadaceae | unclassified | 0 | 2 | 0.31 |  |
| OTU252 | Campylobacteraceae | *Arcobacter* | 2 | 3 | 0.31 |  |
| OTU129 | Moraxellaceae | *Enhydrobacter* | 7 | 0 | 0.32 |  |
| OTU142 | Xanthomonadaceae | *Stenotrophomonas* | 7 | 0 | 0.32 |  |
| OTU24 | Anaerolineaceae | unclassified | 7 | 0 | 0.32 |  |
| OTU60 | Enterobacteriaceae | *Serratia* | 7 | 0 | 0.32 |  |
| OTU68 | Pseudomonadaceae | *Pseudomonas* | 7 | 0 | 0.32 |  |
| OTU54 | Methylocystaceae | unclassified | 7 | 0 | 0.32 |  |
| OTU286 | Xanthobacteraceae | *Variibacter* | 2 | 6 | 0.33 |  |
| OTU258 | Entomoplasmataceae | *Entomoplasma* | 1 | 2 | 0.34 |  |
| OTU113 | Aeromonadaceae | *Aeromonas* | 6 | 0 | 0.35 |  |
| OTU169 | Oxalobacteraceae | *Herminiimonas* | 6 | 0 | 0.35 |  |
| OTU13 | Caulobacteraceae | *Brevundimonas* | 6 | 0 | 0.35 |  |
| OTU158 | Comamonadaceae | *Delftia* | 6 | 0 | 0.35 |  |
| OTU86 | Rhodocyclaceae | unclassified | 6 | 0 | 0.35 |  |
| OTU52 | Gammaproteobacteria | unclassified | 6 | 0 | 0.35 |  |
| OTU63 | Staphylococcaceae | *Staphylococcus* | 6 | 0 | 0.35 |  |
| OTU73 | Enterobacteriaceae | unclassified | 6 | 0 | 0.35 |  |
| OTU77 | Veillonellaceae | *Veillonella* | 6 | 0 | 0.35 |  |
| OTU47 | Nocardiaceae | *Rhodococcus* | 6 | 0 | 0.35 |  |
| OTU194 | Acidobacteriaceae | unclassified | 489 | 847 | 0.35 |  |
| OTU145 | Acidobacteriaceae | unclassified | 15 | 8 | 0.36 |  |
| OTU191 | Nitrosomonadaceae | unclassified | 2 | 5 | 0.36 |  |
